# Advances in Organic Fluorescent Probes for Intracellular Zn^2+^ Detection and Bioimaging

**DOI:** 10.3390/molecules29112542

**Published:** 2024-05-28

**Authors:** Yi Chen

**Affiliations:** 1 Key Laboratory of Photochemical Conversion and Optoelectronic Materials, Technical Institute of Physics and Chemistry, Chinese Academy of Sciences, Beijing 100190, China; yichen@mail.ipc.ac.cn; 2 University of Chinese Academy of Sciences, Beijing 100190, China

**Keywords:** intracellular Zn^2+^, fluorescent probe, detection, imaging

## Abstract

Zinc ions (Zn^2+^) play a key role in maintaining and regulating protein structures and functions. To better understand the intracellular Zn^2+^ homeostasis and signaling role, various fluorescent sensors have been developed that allow the monitoring of Zn^2+^ concentrations and bioimaging in live cells in real time. This review highlights the recent development of organic fluorescent probes for the detection and imaging of intracellular Zn^2+^, including the design and construction of the probes, fluorescent response mechanisms, and their applications to intracellular Zn^2+^ detection and imaging on-site. Finally, the current challenges and prospects are discussed.

## 1. Introduction

Zinc (Zn^2+^), proposed as a novel intracellular second messenger [1,2], plays three main roles in living cells: enzyme cofactor [3,4], signaling mediator [5,6,7], and protein structural modulator [8,9]. It is reported that more than three thousand proteins require Zn^2+^ to function properly [10,11]. Recently, a new Zn^2+^-associated signal transduction, zinc sparks, was observed [12,13], and a large body of evidence suggests that Zn^2+^ is pivotal to autophagy [14,15,16,17].

The concentration of labile Zn^2+^ varies in different environments. Labile Zn^2+^ in serum or plasma is, for example, approximately 1−10 μM [18,19], while in the cytosol, labile Zn^2+^ is controlled between 100 pM and 1 nM [20,21,22]. A deficiency or excess of Zn^2+^ can cause diseases, and some diseases such as compromised immunity, impaired cognition, and abnormal brain development are believed to be related to Zn^2+^ deficiency [23,24]. On the other hand, Zn^2+^ overabundance leads to cytotoxicity [25,26,27], gastrointestinal discomfort, and disrupted cholesterol metabolism. Moreover, the disruption of Zn^2+^ homeostasis can potentially disturb other transition metal ions’ homeostasis [28]. Therefore, quantitative clarification of Zn^2+^ dynamics at the organelle level can provide fundamental information to better understand the physiological roles of Zn^2+^ in individual organelles and assist in elucidating the networks of Zn^2+^ in cells [29,30].

Fluorescent probes are widely used to investigate biomolecules in living cells, tissues, and animals owing to their high sensitivity and non-invasiveness. Fluorescent Zn^2+^ probes reported mainly include small molecule-based organic chemosensors [31,32,33,34,35,36] and fluorescent protein-based biosensors [37,38,39,40,41]. Compared to fluorescent proteins, small molecule-based organic fluorescent probes have some merits, such as strong emission, tunable wavelength, and more building blocks for probe construction. This review focuses on small molecule-based organic fluorescent probes and summarizes recent progress in fluorescent probes for the detection and imaging of intracellular Zn^2+^.

## 2. Design Strategies for Probes and Their Response Mechanisms

**Scheme 1:**
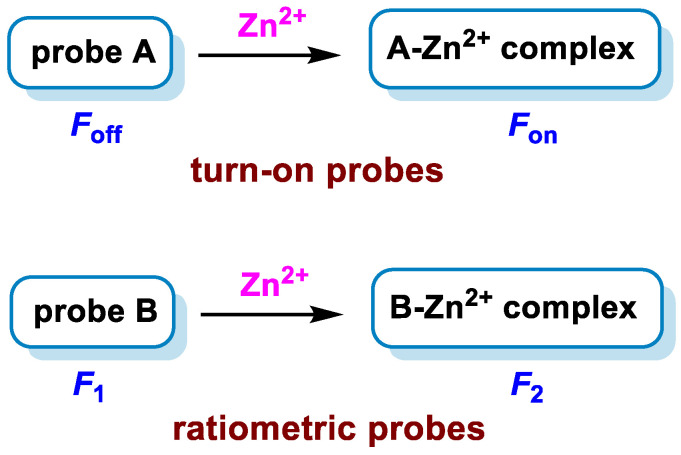
Design strategies for turn-on probes (**upper**) and ratiometric probes (**lower**) for Zn^2+^ detection.

A candidate fluorescent probe for biological application has some merits, including high selectivity, high sensitivity, and high resolution. To obtain high sensitivity and high selectivity, small-molecule-based fluorescent probes are usually designed into two types: turn-on probes and ratiometric probes (Scheme 1). For the former, analyte-binding induces an increase in fluorescence intensity, and for the latter, analyte-binding shifts the emission wavelength.

For turn-on probes [42,43,44], most operate on the principle of photo-induced electron transfer (PET) between the fluorophore and Zn^2+^. In the absence of Zn^2+^, the fluorophore is quenched by PET from the electron-rich chelating group. Upon binding Zn^2+^, the PET between the chelating moiety and the fluorophore is disrupted, leading to an increase in fluorescence emission. Manipulation of the fluorophore and binding motif platform can tune the efficiency of PET; therefore, the magnitude of fluorescence changes with Zn^2+^ binding.

For ratiometric probes [45,46,47], Zn^2+^ binding alters the emission wavelength. The design of ratiometric probes is usually based on intramolecular charge transfer (ICT) or metal–ligand charge transfer (MLCT). Before the addition of Zn^2+^, the probes display fluorescence at λ_1_; upon the addition of Zn^2+^, the ligand and Zn^2+^ form complexes that exhibit fluorescence at λ_2_ due to the ICT or MLCT. The fluorescence intensity at λ_2_ increases while at λ_1_ decreases with the increase in Zn^2+^ until titration saturation. With such probes, fluorescence changes are reported as a ratio of the fluorescence intensity at the two wavelengths. Since this approach minimizes artifacts from cellular movements, sample thickness, and probe concentration, these probes are preferred for accurate quantitative analysis because they provide built-in self-calibration for signal correction by measuring the ratio of the intensity profiles at different wavelengths, and thus more reliable quantitative determination can be achieved.

## 3. Fluorescent Probes for the Detection of Intracellular Zn^2+^

### 3.1. Turn-On Probes

**Scheme 2:**
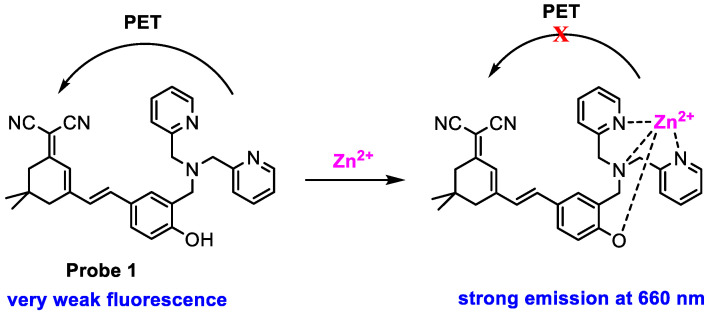
Structure of probe **1** and its response to Zn^2+^.

**Figure 1:**
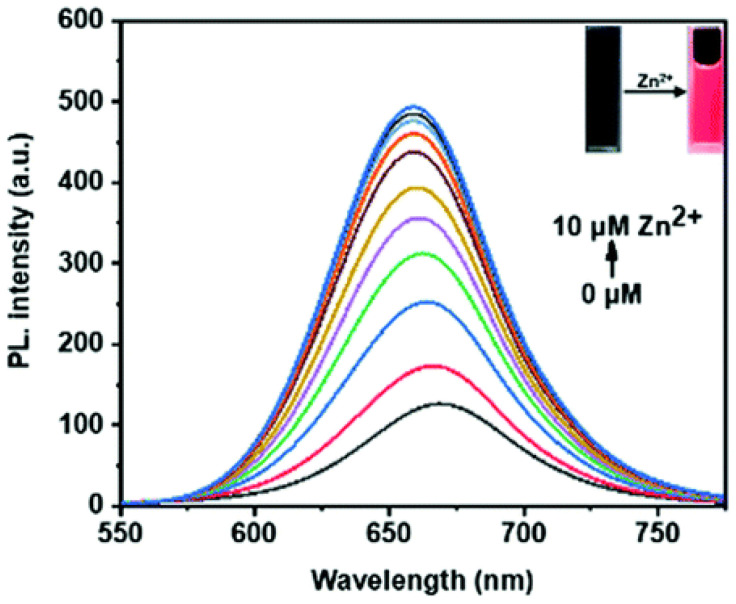
Fluorescence changes in probe **1** response to Zn^2+^ (0–10 μM) in HEPES buffer solution (pH = 7.4) with DMSO (*v*/*v* = 1:1), λ_ex_ = 475 nm, λ_em_ = 660 nm. Reproduced with permission from Ref. [48]. Copyright 2021, The Royal Society of Chemistry.

Feng and co-workers [48] developed a near-infrared (NIR) probe (**1**) (Scheme 2) for monitoring Zn^2+^ in live cells. Probe **1**, synthesized from isophorone in a three-step reaction, displayed a very weak fluorescence at 660 nm (ϕ_f_ = 0.08, HEPES buffer with 50% DMSO, pH 7.4) upon excitation with 475 nm; the low quantum yield resulted from efficient PET from bis(pyridin-2-ylmethyl)amine (electron donator) to 2-(cyclohex-2-en-1-ylidene)malononitrile (electron acceptor). A significantly enhanced fluorescence (ϕ_f_ = 0.44) was obtained when probe **1** was treated with Zn^2+^ due to the block of PET via chelating with Zn^2+^. The changes in fluorescence intensity at 660 nm were proportional to the concentration of Zn^2+^ added over the range tested (Figure 1), and the detection limit was determined to be 0.1 µM. The control experiments demonstrated that probe **1** displayed high selectivity and good pH stability. Little fluorescence change was detected in the physiological pH range (pH = 4–10) and other bio-ions, as well as ROS, RNS, and RSS species.

**Figure 2:**
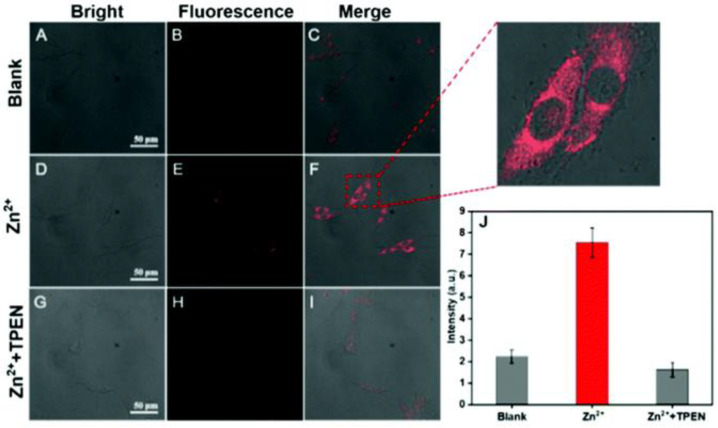
Confocal imaging in PC12 cells. (**A**–**C**) PC12 cells incubated with probe **1** (10 μM) for 30 min. (**D**–**F**) PC12 cells incubated with Zn^2+^ (10 μM) for 10 min, followed by incubation with probe **1** (10 μM) for 30 min. (**G**–**I**) PC12 cells incubated with TPEN (10 μM) for 10 min and with Zn^2+^ (10 μM) for 10 min, followed by incubation with probe **1** (10 μM) for 30 min. λ_ex_ = 561 nm and obtained at 600–700 nm. (**J**) Average intensity of PC12 cells. Error bars express the standard deviation (±S.D.) with *n* ≥ 3. Reproduced with permission from Ref. [48]. Copyright 2021, The Royal Society of Chemistry.

In vitro fluorescence imaging of probe **1** was performed in PC12 cells (a type of nerve cell derived from rat adrenal medullary pheochromocytoma) by treatment with exogenous Zn^2+^. Compared with the controls (Figure 2B,C), the images of PC12 cells showed a significant red fluorescence when they were treated with Zn^2+^ first, followed by incubation with probe **1** (Figure 2E,F). To verify that the red fluorescence resulted from the probe **1** response to Zn^2+^, *N,N,N*′*,N*′-tetrakis(2-pyridylmethyl) ethylenediamine (TPEN), a Zn^2+^ chelator, was used for the control. It was found that the red fluorescence completely faded when the TPEN was added to the PC12 cells (Figure 2H,I). The results suggest that probe **1** can detect an instantaneous change in Zn^2+^ in living cells.

**Figure 3:**
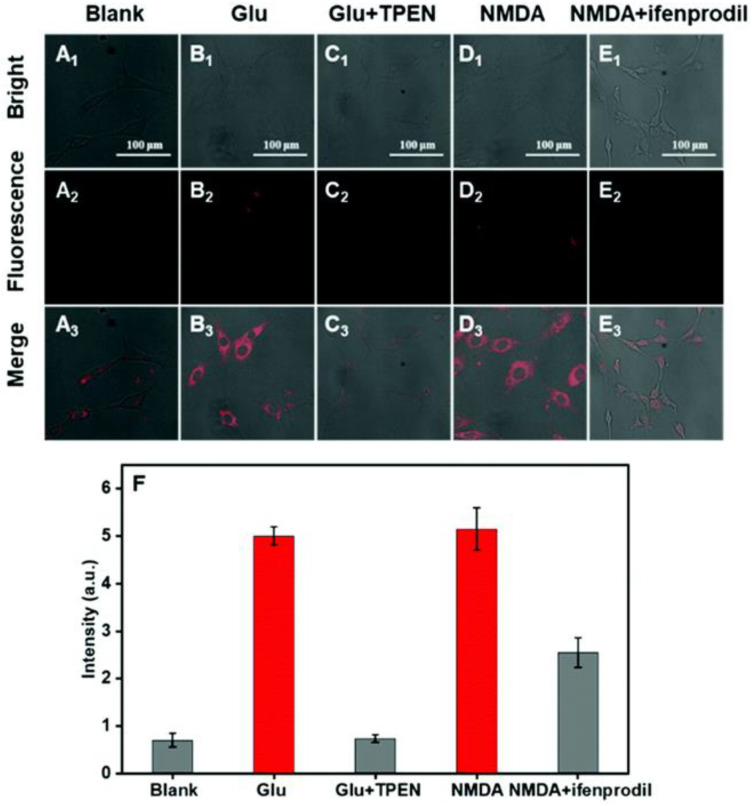
Confocal imaging in PC12 cells. (**A1**–**A3**) PC12 cells incubated with probe **1** (10 μM) for 30 min. (**B1**–**B3**) PC12 cells preincubated with glutamate (10 mM) for 7 h, followed by incubation with probe **1** (10 μM) for 30 min. (**C1**–**C3**) PC12 cells preincubated with glutamate (10 mM) for 7 h and with TPEN (10 μM) for 10 min, followed by incubation with probe **1** (10 μM) for 30 min. (**D1**–**D3**) PC12 cells preincubated with NMDA (4 mM) for 7 h, followed by incubation with probe **1** (10 μM) for 30 min. (**E1**–**E3**) PC12 cells preincubated with NMDA (4 mM) and ifenprodil tartrate (4 mM) for 7 h, followed by incubation with probe **1** (10 μM) for 30 min. Excited with λ_ex_ = 561 nm and collected at 600–700 nm. (**F**) Average intensity in PC12 cells. Error bars express the standard deviation (±S.D.) with n ≥ 3. Reproduced with permission from Ref. [48]. Copyright 2021, The Royal Society of Chemistry.

The detection of endogenous Zn^2+^ in PC12 cells with probe **1** was performed by the stimulation of glutamate. A high concentration of glutamate induces oxidative stress in the nervous system [49], followed by an increase in Zn^2+^ in nerve cells [50]. As shown in Figure 3, the glutamate-stimulated PC12 cells exhibited red fluorescence due to the increase in intracellular Zn^2+^. The red fluorescence was completely suppressed when TPEN was added. Further application of probe 1 to monitor Zn^2+^ in PC12 cells during depression was conducted by incubation of N-methyl-D-aspartic acid (NMDA) receptors, which are postsynaptic targets for releasing Zn^2+^. In comparison with the control group, the PC12 cells, preincubated with NMDA, a specific agonist for the NMDA receptor, exhibited apparent red fluorescence (D2 and D3). The red fluorescence was, however, decreased by the addition of ifenprodil tartrate, a specific NMDA receptor antagonist (E2 and E3). The results suggest that the generation of Zn^2+^ is correlated with the over-activation of the NMDA receptor, and probe **1** can monitor variations in Zn^2+^ in real time during depression.

**Scheme 3:**
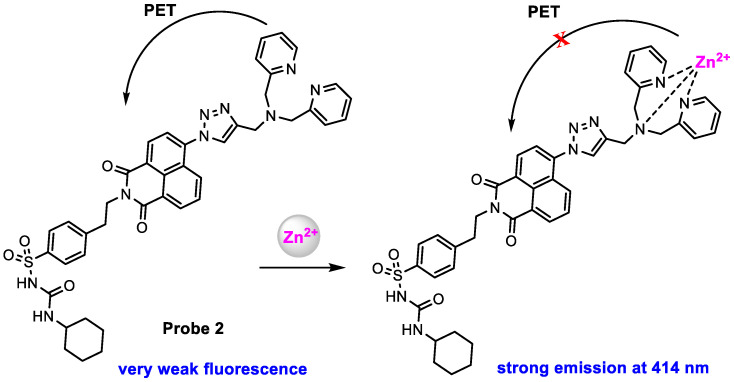
Structure of probe **2** and its response to Zn^2+^.

**Figure 4:**
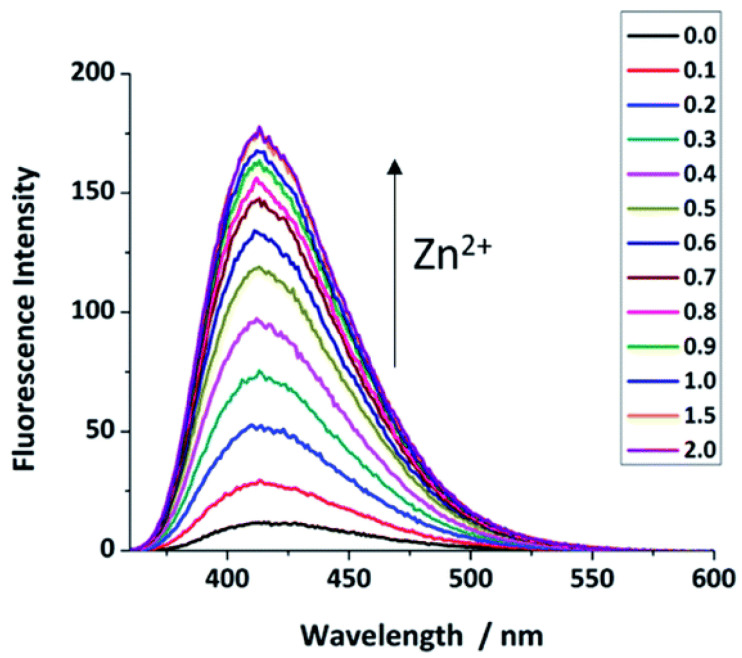
Fluorescence changes in probe **2** response to Zn^2+^ (0–2.0 μM) in HEPES buffer solution (pH = 7.4) with 1% DMSO, λ_ex_ = 346 nm, λ_em_ = 414 nm. Reproduced with permission from Ref. [56]. Copyright 2019, The Royal Society of Chemistry.

Zn^2+^ plays an important role in the function of the endoplasmic reticulum (ER), and its deficiency can cause ER stress [51,52] and result in many diseases [53,54,55]. Fang and co-workers [56] reported an ER-targeted fluorescent probe to image Zn^2+^ in live cells. Probe **2** (Scheme 3) was designed by employing a glibenclamide unit as the ER target and a bis(pyridin-2-ylmethyl)amine unit as the Zn^2+^ chelating agent, both conjugated to 1,8-naphthalic anhydride. Probe **2** exhibited a very weak fluorescence at 414 nm (ϕ_f_ = 0.04, HEPES buffer with 1% DMSO, pH 7.4) with 346 nm excitation. Upon the addition of Zn^2+^, a significantly enhanced fluorescence (ϕ_f_ = 0.25) was observed (Figure 4), and the detection limit was determined to be 47 pM.

**Figure 5:**
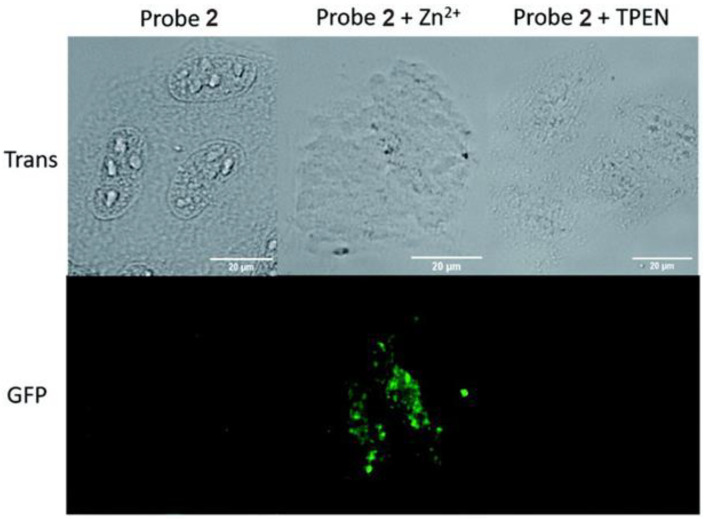
Fluorescence microscopy images of HeLa cells treated with probe **2** (20 µM), probe **2** (20 µM) + zinc pyrithione (100 µM), and probe **2** (20 µM) + TPEN (100 µM). Scale bars: 20 µm. Reproduced with permission from Ref. [56]. Copyright 2019, The Royal Society of Chemistry.

The in vitro imaging of Zn^2+^ in ER with probe **2** was confirmed by the addition of zinc pyrithione, a membrane-permeable zinc source in live cells. As shown in Figure 5, the fluorescence response of probe **2** could be clearly observed in the ER in HeLa cells. Upon the addition of zinc pyrithione, the fluorescence intensity increased significantly; the fluorescence was, however, almost completely quenched by the addition of TPEN.

**Figure 6:**
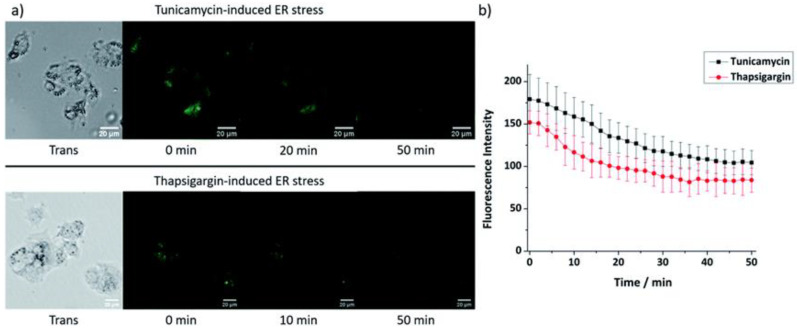
(**a**) Fluorescence microscopy images and (**b**) fluorescence intensity changes in probe **2** (20 µM) in HepG2 cells at ER stress state induced by tunicamycin (5 µg/mL) or thapsigargin (1 µM). Scale bars: 20 µm. Reproduced with permission from Ref. [56]. Copyright 2019, The Royal Society of Chemistry.

The feasibility of probe **2** to monitor changes in Zn^2+^ levels in the ER was carried out with ER stress. ER stress can be induced by tunicamycin or thapsigargin. As shown in Figure 6, the fluorescence intensity of probe **2** in HepG2 cells decreased significantly after treatment with both ER stress inducers, indicating that the mobile Zn^2+^ level in the ER distinctly decreased under ER stress within 1 h. As a control, no fluorescence intensity change was observed in their absence.

**Scheme 4:**
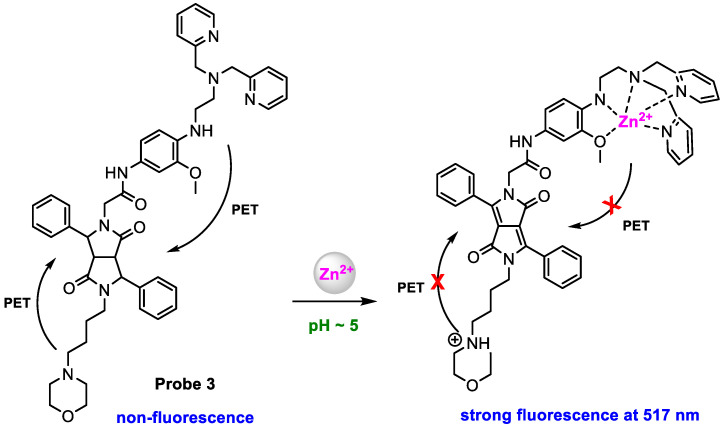
Structure of probe **3** and its response to pH and Zn^2+^.

Du and co-workers [57] synthesized a lysosome-targeted fluorescent probe for in vitro and in vivo imaging of intracellular Zn^2+^. Lysosomes are acidic organelles with a pH of 3.5–6.0, thus, the probe should have dual Zn^2+^ and H^+^ sensing functions. Probe **3** was constructed with three parts: a Zn^2+^ chelator (methoxy-based N,N-di-(2-picolyl)ethylenediamine), a lysosome-targeting moiety (morpholine group), and a fluorophore (3,6-diphenyl diketopyrrolopyrrole). The probe was developed for the detection of low pH and Zn^2+^ in an AND logic fashion (Scheme 4). Enhanced fluorescence was detected when probe **3** (ϕ_f_ = 0.008) was chelated with Zn^2+^ (ϕ_f_ = 0.328) or protonate in morpholine moiety (ϕ_f_ = 0.091), due to the suppression of one PET pathway. A larger increased fluorescence (ϕ_f_ = 0.372) was obtained when probe **3** was chelated with Zn^2+^ at pH = 5; in this case, both PET pathways were blocked.

**Figure 7:**
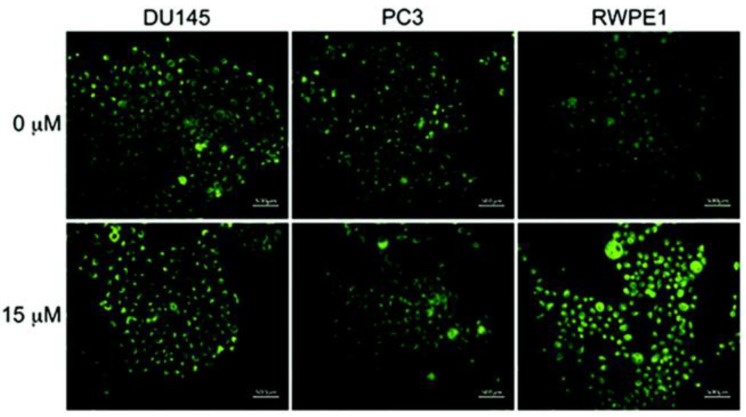
Fluorescence microscope images of cancerous prostate cells (PC3 and DU145) and normal human prostate epithelial cells (RWPE1) incubated with probe **3** (1 µM, 24 h) in the absence and presence of an exogenous Zn^2+^ (15 µM). Reproduced with permission from Ref. [57]. Copyright 2019, The Royal Society of Chemistry.

In vitro imaging of probe **3** was performed by discriminating between cancerous and normal prostate cells. Two prostate cancerous cell lines PC3 and DU145 and one normal human prostate epithelial cell line RWPE1 were employed. No significant change in fluorescence was observed with both cancerous cells DU145 and PC3 with the addition of Zn^2+^ (15 µM). On the contrary, a distinctly enhanced fluorescence was observed with RWPE1 cells with the addition of Zn^2+^ (Figure 7). The difference is ascribed to the downregulation of zinc transporters in cancerous PC3 and DU145 cells, which reduces the uptake of extracellular Zn^2+^.

**Figure 8:**
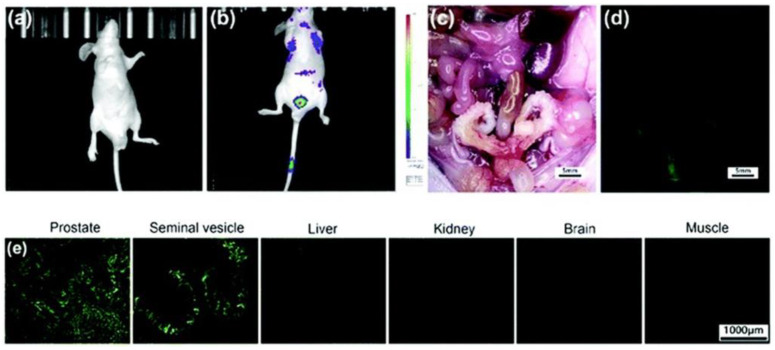
Representative in vivo imaging of tumor-free male BALB/c nude mice before injection (**a**) and after tail-vein injection (**b**) of probe **3** (500 µg per mouse, 5 mice per set). The treated mouse was sacrificed and dissected. The excised prostate and seminal vesicles revealed bright green fluorescence, while other viscera and skeletal muscles remained dark (**c**,**d**). Ex vivo fluorescence imaging of the prostate, seminal vesicles, liver, kidney, brain, and adjacent muscle tissue obtained from the mouse injected with probe **3** (**e**). Reproduced with permission from Ref. [57]. Copyright 2019, The Royal Society of Chemistry.

In vivo imaging of Zn^2+^ with probe **3** was conducted using nude mice models. As shown in Figure 8, a bright fluorescence signal in the region of the prostate was observed (b) after injection of probe **3** (500 µg per mouse) into male BALB/c nude mice via the tail vein for 15 min. The mice were then sacrificed and dissected. Both white-light color images and fluorescent images were taken under the same settings (c,d). The prostate and seminal vesicle displayed a strong green fluorescence, indicating a high concentration of Zn^2+^ in these organs. Furthermore, the prostate and seminal vesicle exhibited a strong green fluorescence signal, whereas little fluorescence was observed in the samples of liver, kidney, brain, and muscle (e).

**Figure 9:**
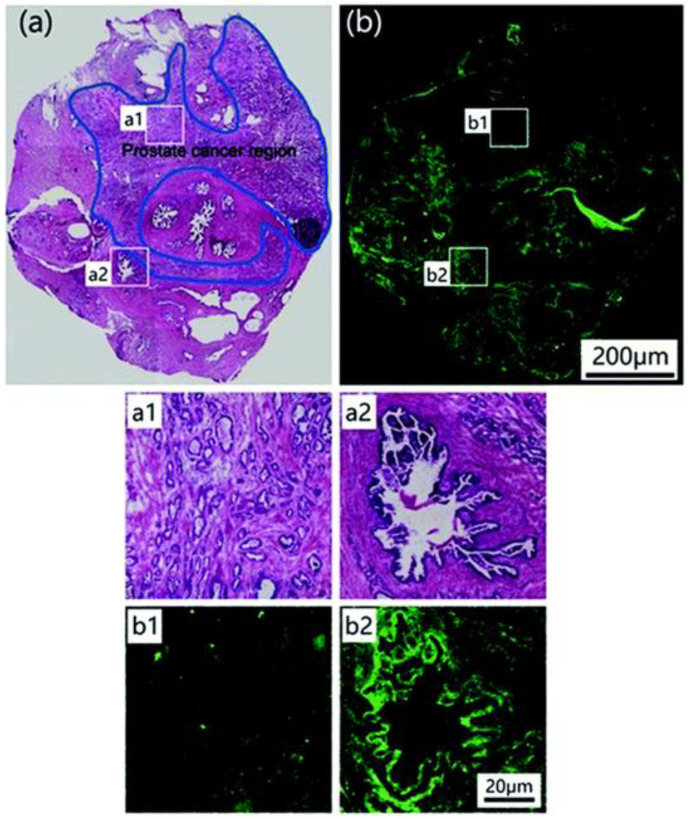
Histological analysis. (**a**) Image of prostate nodule sections stained by H&E staining. The cancerous region is indicated by the blue line. (**b**) Fluorescent staining of the same nodule using probe **3**. Regions of interest (**a1**) and (**b1**) reflect cross-sectional cancerous prostate nodules stained by H&E staining and probe **3**, respectively. Regions of interest (**a2**) and (**b2**) are cross-sectional views of normal prostate nodules stained by H&E staining and probe **3**, respectively. Reproduced with permission from Ref. [57]. Copyright 2019, The Royal Society of Chemistry.

The potential of probe 3 in prostate cancer biomedical imaging was carried out with a prostate nodule obtained from a 65-year-old male patient diagnosed with prostate cancer. H&E staining highlighted the cancerous region (area within the blue line in Figure 9a). Subsequent staining with probe **3** allowed a clear visualization of the cancerous regions as shown by regions of relatively weak fluorescence (Figure 9b). A significant difference in fluorescence intensity between healthy (strong fluorescence) and cancerous tissue (weak fluorescence) was observed. Authors propose that probe **3** may have a role to play in identifying cancerous prostate tissue in human samples.

**Scheme 5:**
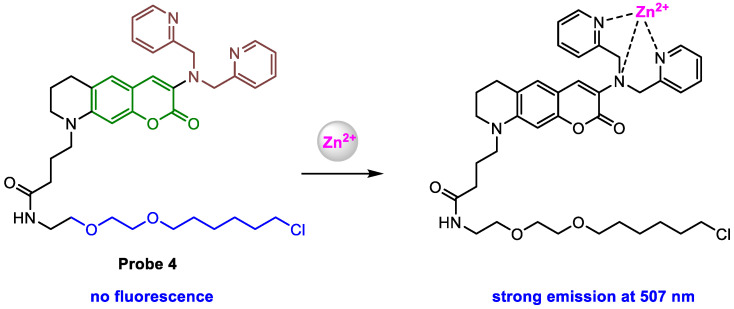
Structure of probe **4** and its response to Zn^2+^.

Kowada and co-workers [58] employed HaloTag technology [59], a protein-labeling technology, to localize fluorescent probes to a specific subcellular compartment [60,61,62], to target probe **4** (Scheme 5) into the Golgi apparatus. Probe **4** was constructed with four parts: a fluorophore (green color), a Zn^2+^ chelator (brown color), a HaloTag ligand (blue color), and a linker (black color). Probe **4** showed almost no fluorescence before the response to Zn^2+^, but a strong fluorescence (λ_em_ = 507 nm, ϕ_f_ = 0.52) was obtained upon the addition of the Zn^2+^. Fluorometry revealed that probe **4** exhibited Zn^2+^-dependent enhancement of fluorescence. The control experiments suggested that probe **4** displayed high selectivity to the detection of labile Zn^2+^ without interference from other metal ions. In addition, probe **4**, together with its Zn^2+^-complex, exhibited almost constant fluorescence intensity in the pH range from 6 to 8.

**Figure 10:**
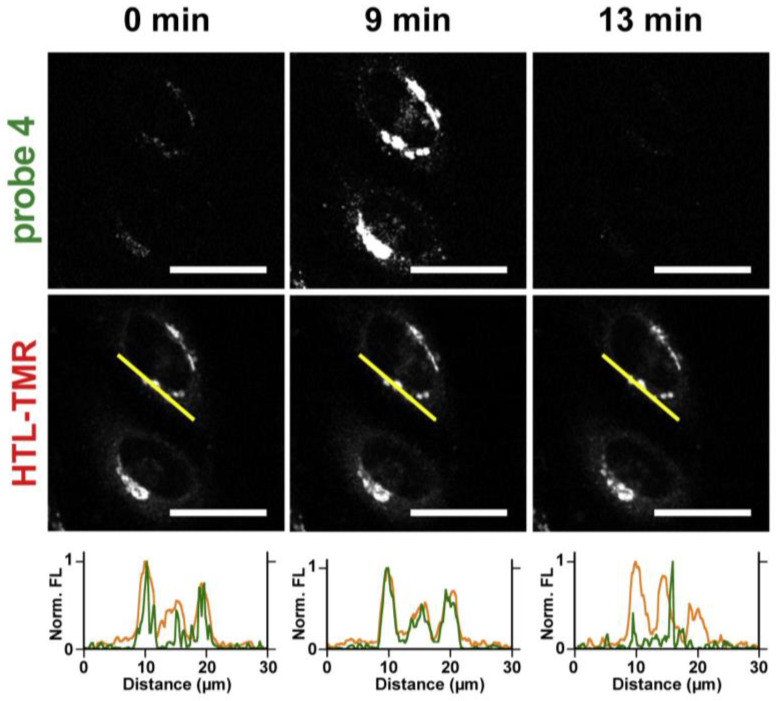
Confocal microscopy images of probe **4** (0.25 µM) and HTL-TMR (5.0 nM) at 0 min (steady-state), 9 min (saturation), and 13 min (depletion). The line profiles indicate the fluorescence intensities of probe **4** (green) and HTL-TMR (orange), measured along the yellow lines in the HTL-TMR images. Scale bar: 30 mm. Zn^2+^/pyrithione (ZPT, 40 µM/2 µM, at 2 min) and TPEN (80 µM, at 10 min) were added to increase and decrease intracellular Zn^2+^. Reproduced with permission from Ref. [58]. Copyright 2020, Elsevier.

Monitoring the concentration change in Zn^2+^ with probe **4** in the Golgi apparatus was conducted by using a co-localizable internal standard dye, HaloTag TMR ligand (HTL-TMR), for in situ standard quantification. Probe **4** and HTL-TMR were co-incubated with HeLa cells at a ratio of 50:1. As shown in Figure 10, significant fluorescence was detected when Zn^2+^/pyrithione (ZPT) was added to the cells, and the fluorescence then decreased when TPEN was subsequently added. The results suggest that probe **4** can be used to visualize the change in Zn^2+^ in the Golgi.

**Figure 11:**
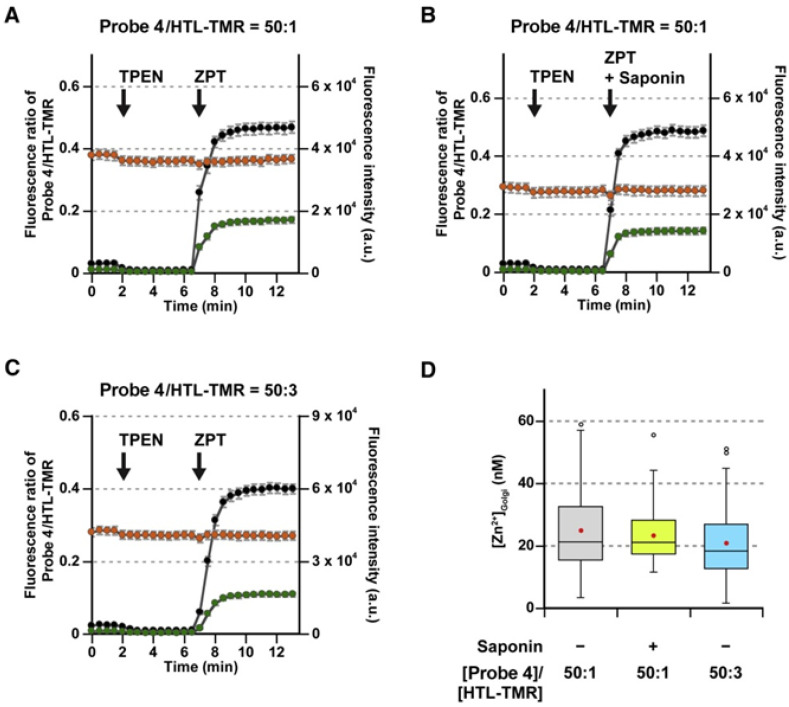
Quantitative imaging of Zn^2+^ in the Golgi apparatus in live HeLa cells with a combination of probe **4** and HTL-TMR. (**A**–**C**) Time course of fluorescence ratios (black) and intensities of probe **4** (green) and HTL-TMR (orange). The HaloTag proteins were labeled with 0.25 µM probe **4** and HTL-TMR at a ratio of 50:1 (**A**,**B**) or 50:3 (**C**). For intracellular Zn^2+^ calibration in live cells, 10 µM TPEN was added at 2 min. ZPT (400 µM Zn^2+^/2 µM pyrithione) (**A**,**C**) or ZPT (400 µM Zn^2+^/2 µM pyrithione) with 0.002% saponin (**B**) was then added at 7 min. Error bars represent SEM ((**A**): n = 106, N = 9; (**B**): n = 49, N = 4; (**C**): n = 104, N = 9). n and N indicate the numbers of the examined cells and the independent experiments, respectively. (**D**) [Zn^2+^]Golgi at the steady state in HeLa cells. Plots were constructed from the datasets in Figure 12A–C. Red dots represent the mean values. Reproduced with permission from Ref. [58]. Copyright 2020, Elsevier.

Quantitative analysis of Zn^2+^ in the Golgi was based on the ratio value of the fluorescence intensities of probe **4** and HTL-TMR. The concentration of Zn^2+^ in the Golgi was estimated in the range of 3.3–59 nM, and the average value was calculated to be 25 ± 1 nM (mean ± SEM). Further verification was carried out by using saponin (a membrane-permeabilizing agent) to facilitate the intracellular Zn^2+^/pyrithione influx as the control (Figure 11B), and the resulting concentration of Zn^2+^ was determined to be 23 ± 1 nM (Figure 11D).

**Scheme 6:**
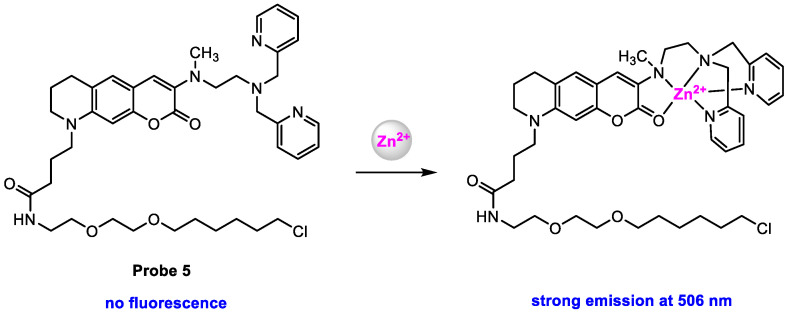
Structure of probe **5** and its response to Zn^2+^.

To obtain the quantification of Zn^2+^ in various organelles, the same group [63] modified probe **4** with a higher-affinity Zn^2+^-binding motif to increase the Zn^2+^ affinity of the probe. Probe **5** (Scheme 6) was designed to increase the Zn^2+^ affinity by increasing the coordination number of the chelator moiety. Compared to probe **4** (dissociation constant: *K*_d_ = 240 nM), probe **5** exhibited a much higher affinity for Zn^2+^ (*K*_d_ = 0.16 nM) in HEPES buffer (pH = 7.4).

Probe **5** showed very weak fluorescence due to PET before the response to Zn^2+^, but strong fluorescence (λ_em_ = 506 nm, ϕ_f_ = 0.57) was obtained upon the addition of Zn^2+^, and fluorescence intensity at 506 nm increased with increasing the concentration of Zn^2+^. Probe **5** exhibited less change in fluorescence in the pH range from 5.5 to 8.0, which suggested that probe **5** could be applied for the detection of Zn^2+^ in various organelles such as the mitochondria, ER, nucleus, and cytosol. The control experiments demonstrated that probe **5** displayed high selectivity to monitor the intracellular Zn^2+^ without interference from other bio-ions.

**Figure 12:**
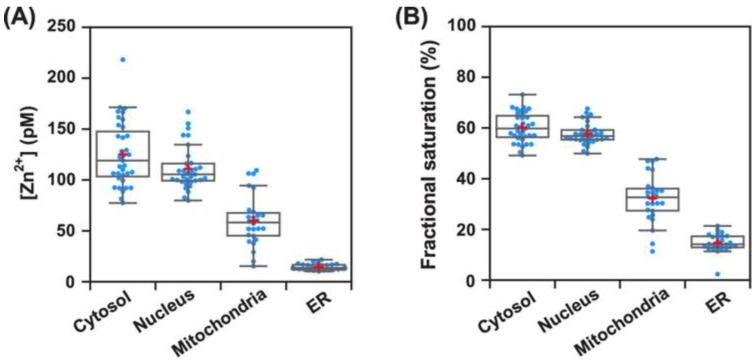
Quantitative mapping of intracellular Zn^2+^ using probe **5** (**A**) and fractional saturation of probe **5** at the steady state (**B**) in various organelles. Red plus symbols represent the mean values. Reproduced with permission from Ref. [63]. Copyright 2022, American Chemical Society.

To obtain the concentration of Zn^2+^ in various organelles, probe **5** was precisely localized to the target cellular compartments including the mitochondria, ER, nucleus, and cytosol using the HaloTag labeling technology. The quantification of Zn^2+^ in various organelles was performed based on the in situ standard quantification method via co-labeling with HTL-TMR. With Halo−probe **5** at pH 7.4, the concentrations of Zn^2+^ in various organelles were estimated to be 0.13 nM (cytosol), 0.11 nM (nucleus), 14 pM (ER), and 60 pM (mitochondria), respectively (Figure 12A). In all the tested organelles, the fractional saturation values of probe **5** were in the range of 10−90% (Figure 12B), indicating that probe **5** is appropriate for the quantification of Zn^2+^.

### 3.2. Ratiometric Probes

Two-photon (TP) microscopy is an important tool for biological imaging due to some merits, such as excitation at NIR wavelengths, refraining from light scattering, and providing high-resolution imaging. These promising features have motivated the search for two-photon fluorescent probes for the detection and imaging of biological analytes [64,65,66,67,68].

**Scheme 7:**
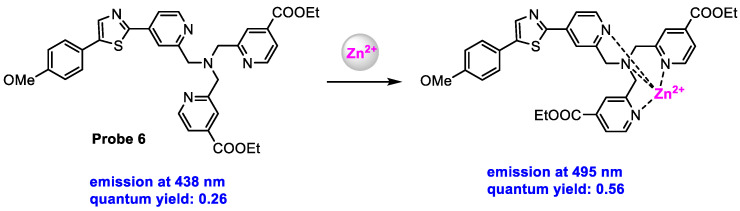
Structure of probe **6** and its response to Zn^2+^.

Bourassa and co-workers [69] synthesized a TP-induced ratiometric fluorescent probe for imaging of dynamic changes in labile Zn^2+^ in oligodendrocytes, an important cellular constituent of the brain, at different stages of development, in a cell culture. Probe **6** (Scheme 7) displayed fluorescence at 438 nm with a quantum yield of 0.26 in a neutral aqueous buffer (pH 7.0, 10 mM PIPES 0.1 M KCl). After binding with Zn^2+^, a Zn^2+^-complex with 1:1 metal−ligand stoichiometry was obtained, which showed fluorescence at 495 nm with a quantum yield of 0.56.

**Figure 13:**
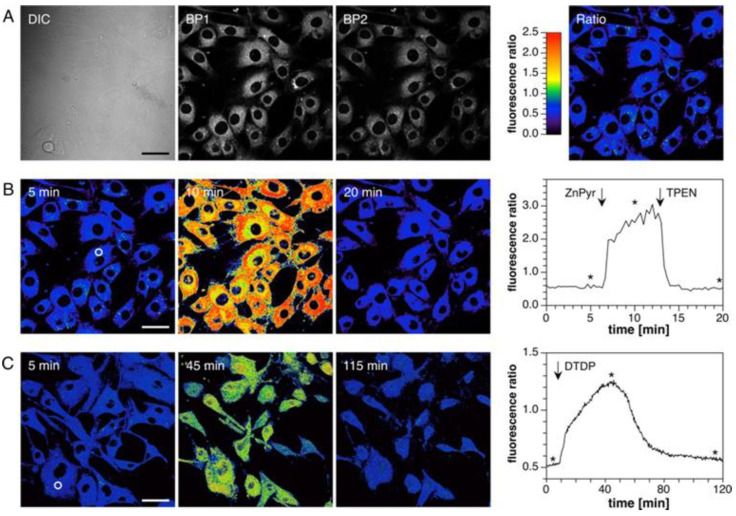
Ratiometric imaging of Zn^2+^ in live NIH 3T3 mouse fibroblasts with probe **6** by TP excitation at 720 nm. (**A**) Left: DIC and fluorescence images acquired with 425−462 nm (BP1) and 478−540 nm (BP2) bandpass filters, respectively. Right: Intensity ratio image with R = BP2/BP1. (**B**) Left: Ratio images (BP2/BP1) at selected time points prior to and after addition of 50 μM ZnSO_4_ and 5 μM pyrithione (ZnPyr). Further addition of 100 μM TPEN at 13 min resulted in a reversal of the intensity ratio (shown at 20 min). Right: Time course of the average intensity ratio change for the ROI indicated with a white circle in the left panel. The asterisks indicate time points for the respective ratio images shown to the left. (**C**) Left: Ratio images prior (at 5 min) and after addition of 100 μM DTDP (at 45 min). This treatment resulted first in a ratio increase peaking around 40 min followed by a gradual decrease back to the initial level (shown at 115 min). Right: Time course of the average intensity ratio change for the ROI indicated with a white circle. The asterisks indicate time marks for the respective ratio images to the left. Scale bars: 40 μm. Reproduced with permission from Ref. [69]. Copyright 2018, American Chemical Society.

Ratiometric imaging of cellular Zn^2+^ was performed with NIH 3T3 mouse fibroblasts as a model system. Live cells were incubated with probe **6** (10 μM) and imaged by TP excitation at 720 nm. Fluorescence was measured between 425–462 (BP1) nm and 478–540 nm (PB2) (Figure 13A), respectively, and the corresponding ratio image was obtained with an average ratio R (PB2/PB1) of 0.60 ± 0.16. Upon exposure to a mixture of ZnSO_4_ and pyrithione, R was increased to 2.53 ± 0.56, indicating saturation of the probe with Zn^2+^. The addition of chelator TPEN decreased R to 0.54 ± 0.13 (Figure 13B, left). A plot of the intensity ratio vs. time revealed rapid dynamics for Zn^2+^-binding and release (Figure 13B, right). Dynamic fluctuations in endogenous Zn^2+^ were also imaged with probe **6**. 2,2′-dithiodipyridine (DTDP), an oxidizing agent that induces dissociation of Zn^2+^ from cellular sulfhydryl binding sites [70], was employed to incubate with the cells. A low R (0.5) was obtained, which increased to 1.3 upon the addition of DTDP (100 μM). The increased R was then decreased back to basal conditions (R = 0.5) with the increase in time due to the lack of exogenous Zn^2+^ (Figure 13C).

**Figure 14:**
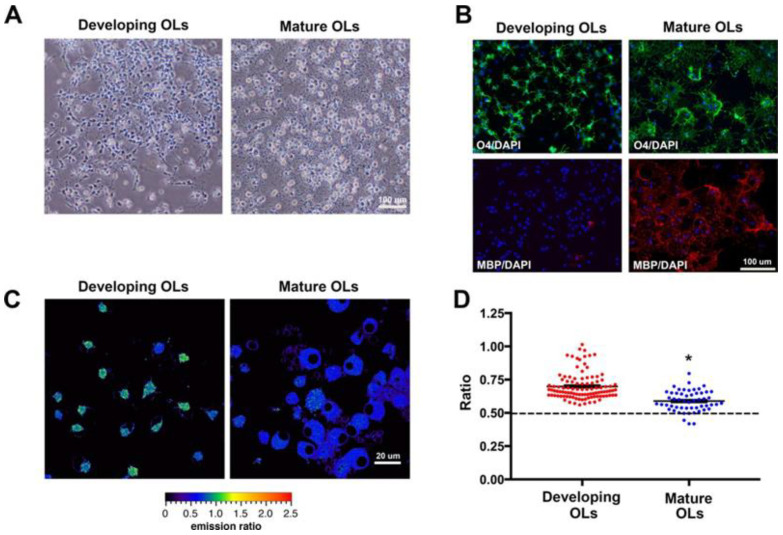
(**A**) Phase contrast microscopy images of developing oligodendrocytes at DIV9 and mature oligodendrocytes at DIV16. (**B**) Immunocytochemistry for O4 antigen (O4, green), myelin basic protein (MBP, red), and DAPI (blue) in developing and mature oligodendrocytes. (**C**) Representative probe **6** ratio images for developing and mature oligodendrocytes at DIV9 and DIV16, respectively. (**D**) Scatter plot for probe **6** ratios of individual developing (red) and mature (blue) oligodendrocytes. Mean ± SEM, shown with black solid lines, indicated a decreased ratio in mature oligodendrocytes compared to developing oligodendrocytes. * *p* < 0.0001 (*t*-test developing vs. mature oligodendrocytes). The broken line indicates the average ratio after application of TPEN. Reproduced with permission from Ref. [69]. Copyright 2018, American Chemical Society.

Probing labile Zn^2+^ in developing oligodendrocytes with probe **6** showed that the concentrations of intracellular Zn^2+^ in oligodendrocytes at different stages of development were different. O4 antigen (O4) and myelin basic protein (MBP) were employed as a marker of developing and mature oligodendrocytes, respectively, and both representative phase contrast images and fluorescence immunohistochemistry images were obtained (Figure 14A,B). As expected, there was no MBP expression in the developing oligodendrocytes but robust expression in mature oligodendrocytes. Similar to cultures with probe **6**, as shown in Figure 14C,D, the intensity ratio of images was decreased in mature (0.59 ± 0.01, n = 54 cells from three different cultures) versus developing (0.70 ± 0.01, n = 103 cells from three different cultures) oligodendrocytes.

**Scheme 8:**
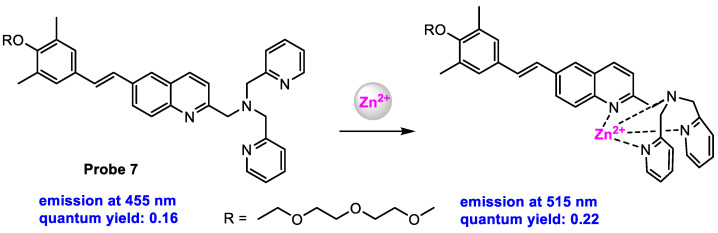
Structure of probe **7** and its response to Zn^2+^.

Li and co-workers [71] reported a TP-induced ratiometric fluorescent probe for in vitro and in vivo imaging of Zn^2+^. Probe **7** (Scheme 8) displayed fluorescence at 455 nm with a moderate quantum yield of 0.16 in HEPES buffer containing 10% MeOH (pH 7.4). Upon addition of Zn^2+^ to the solution of the probe, a 1:1 stoichiometric Zn^2+^-complex was obtained, which showed fluorescence at 515 nm with a quantum yield of 0.22. The control experiment confirmed that the developed probe showed high selectivity for fluorescent responses to Zn^2+^. The limit of detection (LOD) was calculated at 25 ± 5 nM.

**Figure 15:**
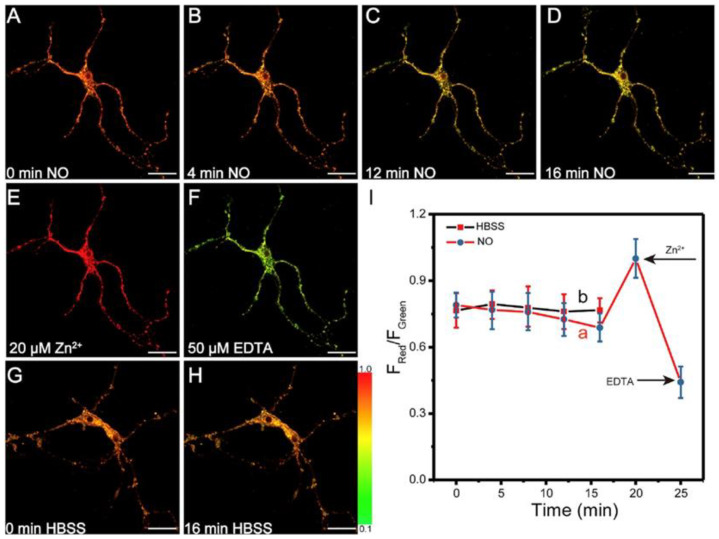
TP fluorescent images of probe **7** (5 μM) with NO in neurons. Ratiometric imaging of Zn^2+^ in live NIH 3T3 mouse fibroblasts with probe **6** by TP excitation at 720 nm. (**A**–**D**) Fluorescence images of probe **7** incubated with NO for 0 min, 4 min, 12 min, and 16 min, (**E**) subsequently treated with Zn^2+^/pyrithione (10 μM, 1:2), and (**F**) followed by treatment with EDTA (20 μM). (**G**,**H**) Fluorescence images of neurons treated with HBSS for 0 and 16 min; scale bars: 15 μm. (**I**) Quantification of NO-induced (a) and HBSS-treated (b) probe 7 fluorescence changes in neurons over 16 min. After 16 min, the NO-induced sample was treated with 10 μM Zn^2+^/pyrithione (1:2) and followed with 20 μM EDTA (error bars: S.D., n = 10). Reproduced with permission from Ref. [71]. Copyright 2020, Elsevier.

In vitro imaging with probe **7** was evaluated using mouse cortical neurons as a model system by TP excitation at 750 nm. Two emission channels, *F*_green_ (380–470 nm) and *F*_red_ (470–650 nm), were employed for recording, and endogenous Zn^2+^ was obtained by NO-inducing metallothioneins [72]. As shown in Figure 15A–D, the color of the images changed from orange to yellow after the cells were incubated with NO for 16 min, accompanied by the decrease in fluorescent intensity ratio *F*_red_/*F*_green_ from 0.79 ± 0.06 to 0.68 ± 0.07, which resulted from the decrease in the concentration of Zn^2+^ released from the mouse cortical neurons due to the decrease in NO. The control experiments demonstrated that probe **7** exhibited significant reversibility and good photostability. The former was proven by subsequently treated with Zn^2+^/pyrithione followed with EDTA (chelator for Zn^2+^) (Figure 15E,F), the latter by irradiation of probe **7** in HBSS buffer for 16 min (Figure 15G,H).

**Figure 16:**
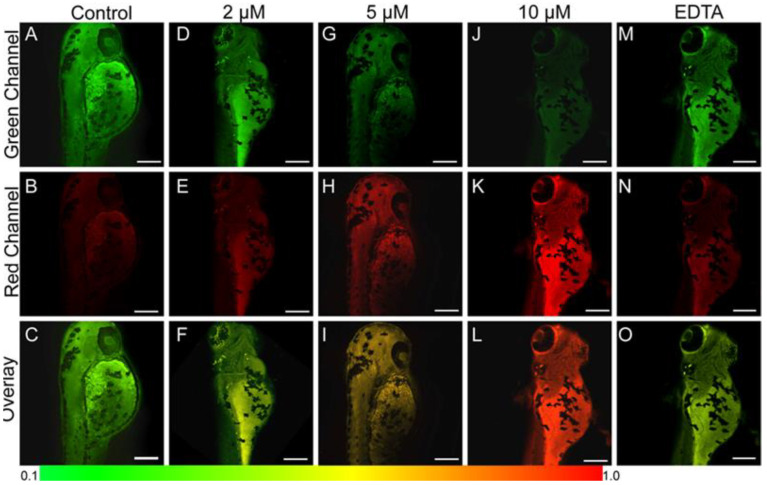
TP fluorescence images of 1-day-old larval zebrafish treated with 5 μM probe **7** in different concentrations of Zn^2+^: (**A**–**C**) 0 μM, (**D**–**F**) 2 μM, (**G**–**I**) 5 μM, (**J**–**L**) 10 μM, and then incubated with 20 μM EDTA (**M**–**O**). Green channel (**A**,**D**,**G**,**J**,**M**) obtained from 380–470 nm, red channel (**B**,**E**,**H**,**K**,**O**) obtained from 470–650 nm, overlay channel (**C**,**F**,**I**,**L**,**O**) represented *F*_red_/*F*_green_; scale bars: 120 μm. Reproduced with permission from Ref. [71]. Copyright 2020, Elsevier.

In vivo TP imaging of probe **7** was evaluated using larval zebrafish as the model. With the increase in Zn^2+^, the color changed from yellow-green to orange, and then to red (Figure 16F–L), accompanied by an increase in fluorescent intensity ratio *F*_red_/*F*_green_ from 0.17 ± 0.15 to 0.91 ± 0.21. After EDTA was added to the larval zebrafish, the color changed back to green (Figure 16O), and the fluorescent intensity ratio *F*_red_/*F*_green_ decreased to 0.21 ± 0.10, due to Zn^2+^ chelation with EDTA.

## 4. Conclusions and Outlook

Since Zinquin was used for investigation of intracellular Zn^2+^ in 1993 [73], great efforts have been made to develop small-molecule fluorescent probes for the dynamic detection of intracellular Zn^2+^. Small-molecule probes are typically not suitable for the time-lapse monitoring of Zn^2+^ dynamics at the organelle level because they quickly diffuse in the cell and often leak from the cell to the medium [74]. To address this shortcoming, targeted small-molecule probes are designed by incorporation of the fluorophore with a targeting group, including triphenylphosphonium [75,76,77], glibenclamide [78,79], and morpholine [80,81]; the resulting probes can selectively localize to specific organelles such as the mitochondria, endoplasmic reticulum, and lysosome, respectively. Recently, naphthalimide-based probes have been reported to selectively localize to particular organelle membranes by adjusting their lipophilicity [82].

Although significant progress in small-molecule fluorescent probes for the detection of intracellular Zn^2+^ has been achieved, most probes are based on the PET mechanism or MLCT mechanism. It should be noticed that PET often exhibits incomplete fluorescence quenching that limits the performance of the probes. Additionally, metal ion binding typically induces a small spectral shift [83,84], which is not good for ratiometric detection since serious overlap between two emission wavelengths can lead to significant errors in results. Therefore, the development of probes with new mechanisms or integration of other mechanisms such as fluorescence resonance energy transfer (FRET) [85,86] and excited-state intramolecular proton transfer (ESIPT) [87,88] in the probe is desired [89,90,91,92,93], since both FRET-based probes and ESIPT-based probes can perform ratiometric detection with a large spectral shift in emission wavelength. For practical applications, several challenges remain. First, the quantitative analysis of Zn^2+^ in various organelles should be clarified. To achieve more accurate quantification of Zn^2+^ in organelles, the probes must fulfill several requirements, including pH insensitivity, good chemical stability, high Zn^2+^-binding affinity, and large dynamic range. Second, for in vivo detection and imaging, NIR or two-photon fluorescent probes have promising potential as a consequence of their low background fluorescent signal and deep penetration depth. Third, high selectivity and sensitivity, as well as fast response, are essential for detection and imaging since organelles exhibit different Zn^2+^ levels and dynamics. In addition, solubility, cell penetrability, and cytotoxicity are important parameters in practical applications, which must be considered in the design of probes.

## Data Availability

Not applicable.
